# Self-Assembled Nanofibrous Membranes by Electrospinning as Efficient Dye Photocatalysts for Wastewater Treatment

**DOI:** 10.3390/polym15020340

**Published:** 2023-01-09

**Authors:** Wafa Shamsan Al-Arjan

**Affiliations:** Department of Chemistry, College of Science, King Faisal University, P.O. Box 400, Hufof 31982, Al-Ahsa, Saudi Arabia; walarjan@kfu.edu.sa

**Keywords:** graphene oxide, eco-friendly solution, polymeric materials, photocatalytic, wastewater treatment

## Abstract

Water pollution has become a leading problem due to industrial development and the resulting waste, which causes water contamination. Different materials and techniques have been developed to treat wastewater. Due to their self-assembly and photocatalytic behavior, membranes based on graphene oxide (GO) are ideal composite materials for wastewater treatment. We fabricated composite membranes from polylactic acid (PLA) and carboxylic methyl cellulose (CMC)/carboxyl-*functionalized* graphene oxide (GO-*f*-COOH) using the electrospinning technique and the thermal method. Then, a nanofibrous membrane (PLA/CMC/GO-*f*-COOH@Ag) was produced by loading with silver nanoparticles (Ag-NPs) to study its photocatalytic behavior. These membranes were characterized using Fourier-transform infrared spectroscopy (FTIR), X-ray diffraction (XRD), scanning electron microscopy (SEM), and transmission electron microscopy (TEM) in order to investigate the behavior of the fabricated membranes. The degradation kinetics studies were conducted using mathematical models, such as the pseudo first- and second-order models, by calculating their regression coefficients (R^2^). These membranes exhibited exceptional dye degradation kinetics. The R^2^ values for pseudo first order were PCGC = 0.983581, PCGC@Ag = 0.992917, and the R^2^ values for pseudo second order were PCGC = 0.978329, PCGC@Ag = 0.989839 for methylene blue. The degradation kinetics of Rh-B showed R^2^ values of PCGC = 0.973594, PCGC@Ag = 0.989832 for pseudo first order and R^2^ values of PCGC = 0.994392, PCGC@Ag = 0.998738 for pseudo second order. The fabricated nanofibrous membranes exhibited a strong π-π electrostatic interaction, thus providing a large surface area, and demonstrated efficient photocatalytic behavior for treating organic dyes present in wastewater. The fabricated PLA/CMC/GO-*f*-COOH@Ag membrane presents exceptional photocatalytic properties for the catalytic degradation of methylene blue (MB) dye. Hence, the fabricated nanofibrous membrane would be an eco-friendly system for wastewater treatment under catalytic reaction.

## 1. Introduction

Poor waste management of toxic substances causes water pollution and poses serious health risks that constitute potential threats to the ecological system. The sharp increase in heavy metals is due to industrial expansion and innovative farming methods [1,2]. Various industrial processes, such as the production of toxic substances, metal plating, tanning, and agrochemicals, lead to increased levels of heavy metal ions in the water. In light of the threat posed by the lack of environmental sustainability to human health and the ability of future generations to live normal lives, it has recently received widespread attention [3,4]. This holds especially true for underwater pollution, which is primarily produced by organic chemicals released by industries that employ chemicals. Photocatalysts exhibit a strong ability to act as catalysts in chemical reactions when heated to standard temperatures and exposed to sunlight [5,6]. The biological decomposition of organic pollutants in water through photocatalysis is a recent development in wastewater treatment. Traditional semiconductor photocatalysts based on TiO_2_ are the primary focus of research in photocatalytic innovation, as well as having several other applications [7,8,9]. Although TiO_2_ has many advantages, such as possessing a variety of sources and a low cost, it is only able to use the ultraviolet portion of the sun [9]. The ultraviolet range is constrained in terms of its practicality, because it only makes up 5% of solar energy. The main aim of current research is to find a catalyst that is able to transform visible light, which makes up over 43% of solar energy. Not only does the inter-band transition in silver nanoparticles (Ag-NPs) cause them to absorb a significant amount of ultraviolet (UV) light, it is also well known that Ag-NPs have several other applications [10,11]. However, the surface plasmon resonance (SPR) factor also allows them to function in visible light. Consequently, unique porous TiO_2_-Ag nanomaterials should be noted; silver nanoparticles (NPs) are promising photocatalysts that function across the solar photovoltaic spectrum [12,13]. They have been demonstrated to possess enhanced visible light photocatalytic activity, resulting in a reduction in various water-soluble dyes and improved water splitting.

In wastewater treatment, nanofiber-based composite materials benefit from easy recycling and electrospinning when producing nanofibrous materials. The many other exceptional characteristics of exfoliated fibrous materials include a particular surface area, good porosity, and the capacity to regulate thickness. The famous graphene derivatives graphene oxide (GO) and reduced graphene oxide (rGO), which have exceptional properties and numerous applications, are well-known 2D materials [14,15]. Due to these properties, electrospun fibers can be used as nanocatalysts in GO-based nanofibrous membranes [16,17,18]. Their controlled dispersibility, many functional oxygen-containing groups, and desired active sites for specialized chemical modifications have generated significant interest.

Furthermore, Ag-NPs exhibit desirable stability with fibrous partners, including GO sheets, and perform better under visible light conditions. Due to the π-π stacking [19] and the electrostatic [20] and weak interaction forces [21] caused by several functional groups, they may be able to separate and adsorb organic dyes [22]. Since GO sheets behave negatively, they may interact with organic matter due to the strong electrostatic interactions caused by carboxylic groups [21,23]. Additionally, the produced nanofibrous membranes function as an effective catalyst for decomposing organic dyes, and because of their exceptional mechanical qualities, they can be reused following wastewater treatment [24].

This study describes the electrospun technique for producing nanofibrous materials while maintaining the standard operating parameters of electrospinning. AgNO_3_ was reduced using ascorbic acid to produce the silver nanoparticles, which were then immobilized on the nanofibrous surface of the PLA/CMC/GO-*f*-COOH. Fibrous matter consisting of PLA/CMC/GO-*f*-COOH was fabricated via electrospinning and thermal processing. Upon initial development of the PLA/CMC/GO-*f*-COOH system, it was used as a matrix material to fabricate electrospun materials using an electrospinning system. The matrix material possessed a high specific surface area and advantageous chemical and mechanical characteristics. We developed cost-effective and environmentally friendly water treatment systems via electrospinning using water as the solvent instead of using organic solvents. The fabricated nanofibrous membranes are environmentally friendly, cheap, and can be used as a catalyst for the treatment of wastewater.

## 2. Materials and Methods

### 2.1. Materials

Carboxymethyl cellulose (CAS# 21902-250G), polylactic acid (CAS# 38534-5G), Graphene oxide (CAS# 763713-250MG), chloroacetic acid (CAS# 402923-500G), Rhodamine B (Rh-B), methylene blue (MB), Sulfuric acid (CAS# 339741-500ML), potassium permanganate (CAS# 223468-500G) and potassium nitrate (CAS# 221295-500G) were supplied by Sigma Aldrich, Petaling Jaya, Selangor, Malaysia. The hydrogen peroxide (CAS# H1009-100ML) and hydrochloric acid (HCl) were purchased from Aladdin Reagent, Shanghai, China.

### 2.2. Methods

#### 2.2.1. Preparation of Polymeric Matrix Solution

**Figure 1:**
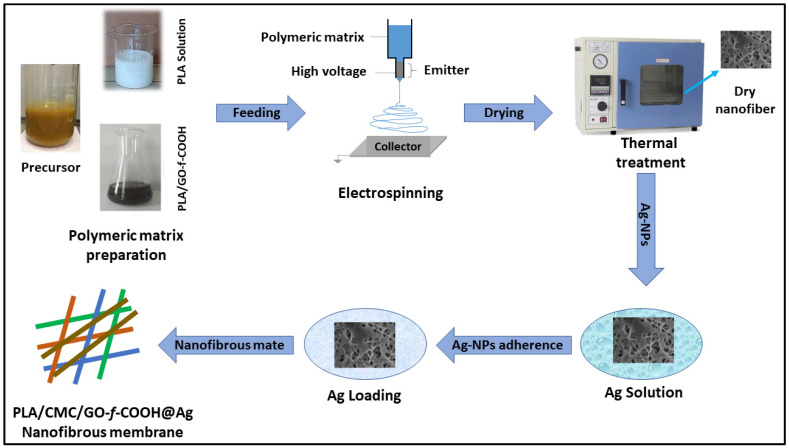
Schematic diagram representing the preparation of a polymeric matrix with which to fabricate nanofibers via electrospinning with thermal treatment, followed by the synthesis of the nanofibrous membrane.

The GO-*f*-carboxyl (GO-*f*-COOH) was synthesized using a well-reported method [25] and freeze dried at a low temperature (−50 °C). PLA solution (10 wt.%) was prepared in deionized water at 80 °C under continuous stirring. CMC solution (30 wt.%) was prepared in deionized water at 35 °C for controlled gelation; otherwise, the occurrence of high gelation may not be conducive to the fabrication of nanofibers. Then, GO-*f*-COOH was added to the CMC solution at 35 °C for 2 h in order to obtain a homogenized solution. The prepared solutions of PLA and CMC/GO-*f*-COOH were mixed in a 1:1 ratio. They were stirred for 2 h in order to obtain a homogenized solution as a polymer matrix solution for fabrication via electrospinning, as shown in Figure 1.

#### 2.2.2. Fabrication of Electrospun Nanofibrous Membrane

First, 10 mL of the polymer matrix solution (i.e., PLA, CMC/GO-COOH, PCGC) was fed into the syringe and fixed on a stainless-steel needle with a diameter of 0.6 mm. The applied voltage difference was 20 kV, with a flow rate of 0.25 mL/h. The distance between the needle and the collector was 20 cm, which was covered by aluminum foil. The nanofibers were collected on the aluminum foil, and the aluminum foil containing nanofibers was placed in an oven for 12 h in order to obtain well-dried nanofibers. The nanofiber samples were placed in a high-temperature oven at 125 °C for 3 h in order to achieve heat-induced cross-linking of hydroxyl functional groups of PLA and carboxylic acid groups CMC/GO-*f*-COOH via esterification reaction, and subsequently coded as PCGC, as shown in Figure 1. Hence, CMC provides desirable gelation [26], PLA enhances the structural integrity [27], GO improves the multifunctional behavior with electroactive behavior due π-π stacking [28], and -COOH provides a highly negative environment for the anchoring of silver nanoparticles [29,30]. The individual behaviors of all of these components have a synergistic catalytic effect on dye degradation that would be helpful in wastewater treatment applications.

#### 2.2.3. Preparation of Nanofibrous Membrane

The fabricated nanofibrous membrane of PCGC was placed into AGNO_3_ (25 mg/mL) under controlled stirring, followed by the addition of ascorbic acid (1 mL) into the AgNO_3_ solution (15 mg/mL). The reduced Ag-NPs slowly started berthing into the insoluble nanofibrous matrix at different time intervals (1, 2, and 5 h), and a treatment with deionized water was performed to eliminate unadhered AG-NPs. The nanofibrous membrane was dried at 80 °C for 24 h in order to obtain a well-dried nanofibrous membrane, which was subsequently coded as PCGC@Ag, as shown in Figure 1.

### 2.3. Characterizations

The structural analysis of fabricated membranes was performed using Fourier-transform infrared spectroscopy (Shimadzu FTIR-8100A, Tokyo, Japan) in the range 4000–400 cm^−1^. Phase identification was performed using X-ray diffraction (Bruker AXS D8 (Kartuizersweg, Kontich, Belgium)) under operating conditions of 45 kV and 45 mA. The morphological analysis of these samples was performed using scanning electron microscopy (JEOLJSM6480, Peabody, MA, USA), and the samples were gold sputtered before SEM analysis with an acceleration voltage of 10 kV. The nanostructure and aggregate of the Ag-NPs were analyzed using an HT7700 transmission electron microscope (Hitachi, Japan). A double-beam UV-visible spectrophotometer (HATCH D500, MA, USA) was used to determine the band gap from absorption spectra to calculate the dye’s concentration in order to investigate dye degradation. The degradation calculations were performed using Equation (1).

(1) $$\mathrm{Degradation}\;\left(\%\right)=\frac{C_{o}-C_{t}}{C_{o}}$$

C_o_ = initial dye conc., and C_t_ = dye conc., at time “*t*.”

### 2.4. Photocatalytic Tests

The dye solutions of Rh-B (5 mg/L) and MB solution (5 mg/L) were prepared in deionized water to determine the photocatalytic activities. The photocatalytic activity of the nanofibrous membrane was determined when added (45 mg) to 100 mL of dye solution and stirred for 30 min under dark conditions until reaching equilibrium. The UV light source was used at a distance of 30 cm to irradiate the liquid surface from the light source. The concentrations of dye solutions were determined at different tine intervals to evaluate the photocatalytic activity of the nanofibrous membrane. The dye solutions were centrifuged to remove the nanocomposite material and supernatant. The calibration curve was drawn, and the degradation (K) was calculated by means of Equation (2).

(2) $$K=\frac{\left(A_{0}-A_{T}\right)}{A_{t}}\times 100\%$$

where *K* = degradation (%); *A*_0_ = original solution absorbance; *A_T_* = solution absorbance at time “*t*” and temperature “*T*.”

## 3. Results and Discussion

### 3.1. Surface Morphology

**Figure 2:**
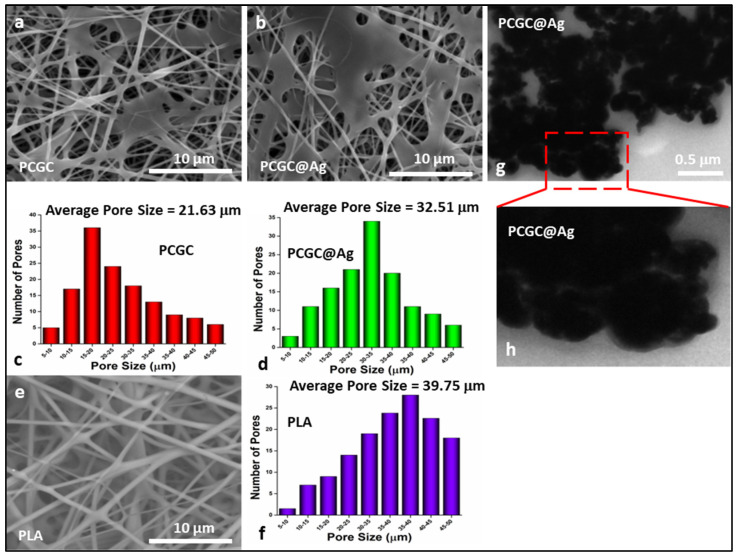
Morphological analysis of the fabricated nanofibrous membranes produced via electrospinning and silver nanoparticles. The SEM morphologies of nanofibers by (**a**) = PCGC, (**b**) = PCGC@Ag, and (**e**) = PLA and their corresponding pore sizes ((**c**) = PCGC, (**d**) = PCGC@Ag, and (**f**) = PLA). The TEM morphologies of the silver nanoparticles on the (**g**,**h**) PCGC@Ag composite nanofibrous membrane samples fabricated using the electrospinning technique, as well as their pore size, were determined by SEM analysis.

The surface morphologies of the nanofibrous membranes were investigated using SEM, as shown in Figure 2a,b,e. The nanostructural morphologies were also represented by SEM. It was observed that the nanofibrous membrane of pure PLA exhibited no clustering of nanocomposite materials on the membranes [31]. However, the nanofibrous membranes possessed nanomaterials, and all of the nanofibrous membranes exhibited uniform fiber diameter distribution sizes of 350–550 nm. The planar structure indicated that successful cross-linking had taken place during thermal treatment, and it was observed that increasing thermal treatment resulted in an increase in the surface area of the planar surface [32,33]. A significant difference in the morphologies of the nanofibrous membranes could clearly be observed, but the fiber diameters were different for all of the nanofibrous materials. The PLA sample had a larger diameter than the PCGC and PCGC@Ag nanofibrous materials, possibly due to the incorporation of the modified natural polymers. The pore size of the nanofibrous materials was in the micrometer range, making them suitable as a potential substrate for the removal of dyes and heavy metals from wastewater. The nanofibrous membrane had a well-distributed pore size, and the PLA sample was more densely fibrous than the other samples.

### 3.2. TEM Analysis

The TEM morphologies of the loaded nanofibrous Ag-NPs were observed, as shown in Figure 2g,h, where it can be seen that the Ag-NPs started to aggregate after one hour. Since the Ag-NPs were synthesized in aqueous solution with several hydroxyl functional groups at the surface of the nanofibers, the microenvironment of the Ag-NP aqueous solution was neutral, and was therefore able to form hydrogen bonds. On the other hand, nanofiber membranes also include many extra carboxyl groups on their surface and can be accepted in large quantities by CMC molecules [32,33]. Therefore, Ag-NPs with numerous hydroxyl groups were readily able to anchor and agglomerate onto the surface of the fabricated nanofibers, primarily because of hydrogen bonding.

### 3.3. FTIR Analysis

**Figure 3:**
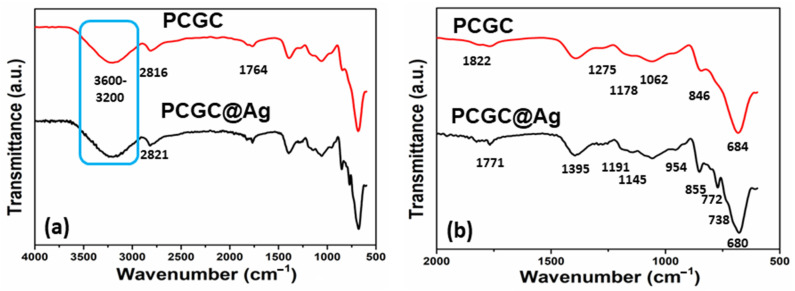
Analysis of the structural and functional groups of the nanofibrous member was performed using FTIR: (**a**) FTIR analysis 4000–500 cm^−1^; and (**b**) FTIR analysis 2000–500 cm^−1^.

The structural and functional groups of PCGC and PCGC@Ag are presented in Figure 3, and the broadband peak at 3600–3200 cm^−1^ is due to the hydroxyl (OH) groups. However, the vibration peaks at 1395 and 1275 cm^−1^ can be attributed to carbonyl (C=O) and hydroxyl (OH) groups [34]. The vibration peaks at 1062 and 1300 cm^−1^ are attributed to cyclic and acyclic (C−O) stretching. However, the vibration peak at 1178 cm^−1^ is an ester (C−O) stretching peak and confirms the formation of an ester bond [35]. The vibration band at 1378 cm^−1^ may be due to the silver nanoparticles anchored on the nanofibrous material. The vibration bands at 2821 and 2816 cm^−1^ are typically saturated aliphatic (C−H) stretching vibrations [36]. The broadband peaks at 3600–3200 cm^−1^ also confirms that CMC and PLA interact due to hydrogen bonding [37]. The hydrogen bonding interaction with the dyes increases with increasing numbers of electronegative atoms and hydrogen-bonding-forming atoms. A strong interaction with metallic nanoparticles is also developed during wastewater treatment. Hence, the analysis confirms that all of the interactions are available and present different interactions, including hydrogen bonding, which is essential for wastewater treatment.

### 3.4. XRD Analysis

**Figure 4:**
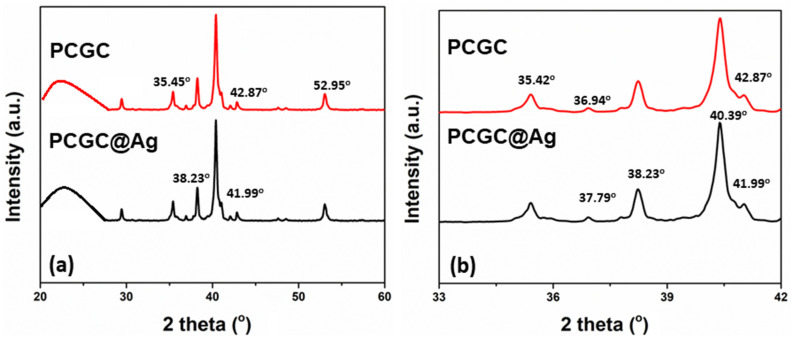
The amorphous and crystalline behavior was determined by XRD: (**a**) with 2θ ranging from 20° to 60°; and (**b**) with 2θ ranging from 33° to 42°.

The diffractogram illustrates the characteristic peak of the Ag-NPs, as shown in Figure 4. X-ray diffraction (XRD) was performed to study the close relationship between silver nanoparticles and nanofibrous membranes. The diffraction patterns of the nanofibrous membranes were modified as a result of their different interactions with the Ag-NP solution. The determined XRD values were observed at 2θ values of 38.23°, 41.99°, 42.87°, and 52.95°, corresponding to Miller indices of (111), (200), (220), and (311), conforming with silver [38,39]. The board peak from 2θ 20 to 30 is due to the polymeric and GO materials, representing the amorphous phase. At the same time, the peaks may correspond to aluminum particles that may adhere after fabrication. Therefore, it is once again confirmed that the nanofibrous membrane contains nanoparticles of silver.

### 3.5. Photocatalytic Efficiency

**Figure 5:**
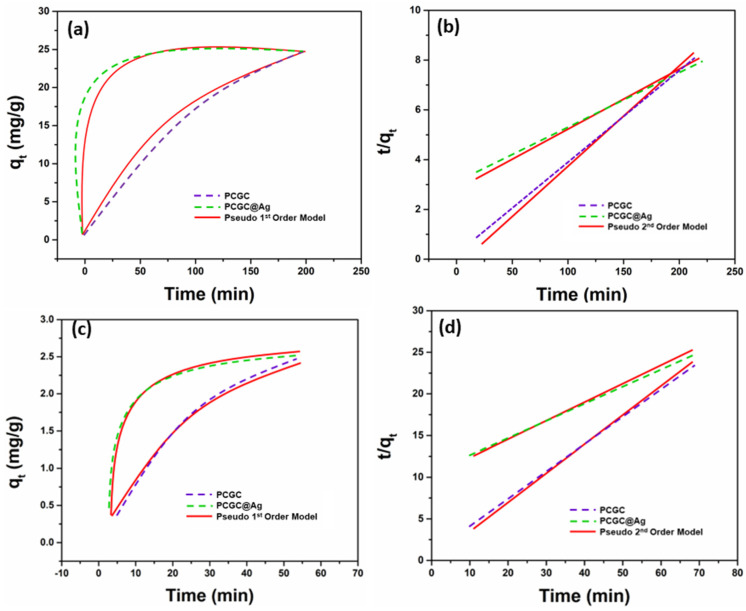
The photocatalytic degradation of model dyes (MB and Rh-B) using kinetics curves of the fabricated nanofibrous membrane PCGC@Ag: MB (**a**,**b**) and Rh-B (**c**,**d**) at 25 °C.

The photocatalytic efficiency of the fabricated PCGC@Ag nanofibrous membrane was evaluated by kinetics studies to determine the dye degradation capacity, as shown in Figure 5a–d. We selected methylene blue (MB) and rhodamine B (Rh-B) to determine the photocatalytic properties of the fabricated material. These are typical model dyes used at a lab scale for wastewater treatment. The photocatalytic behavior was studied three times, and the kinetics model [40] was used to evaluate the degradation kinetics of MB and Rh-B using Equations (3) and (4).

Pseudo first-order model:

(3) $$\log\left(q_{e}-q_{t}\right)=\log q_{t}-\frac{k}{2.303}t$$

Pseudo second-order model:

(4) $$\frac{t}{q_{t}}=\frac{t}{q_{e}^{2}}+\frac{t}{q_{e}}$$

where *q_e_* & *q_t_* = dye degraded quantity at equilibrium and at time “*t*”; and k1 and k2 = kinetic rate constants for pseudo first- and second-order models.

The results of the kinetics analysis obtained for the pseudo first-order and pseudo second-order models are summarized in Table 1. The correlation coefficient values for PCGC and PCGC@Ag were R^2^ > 0.983581 and R^2^ > 0.992917, respectively, for Rh-B. However, the correlation coefficient values for PCGC and PCGC@Ag were found to be R^2^ > 0.973594 and R^2^ > 0.989832, respectively, for MB. The experimental efficiency of the control group (PCGC) of the PLA/CMC/GO-*f*-COOH nanofibrous membrane was better than that of the nanofibrous membrane silver-modified nanofibrous material. However, the kinetics study performance of PCGC@Ag is better than PCGC.

**Figure 6:**
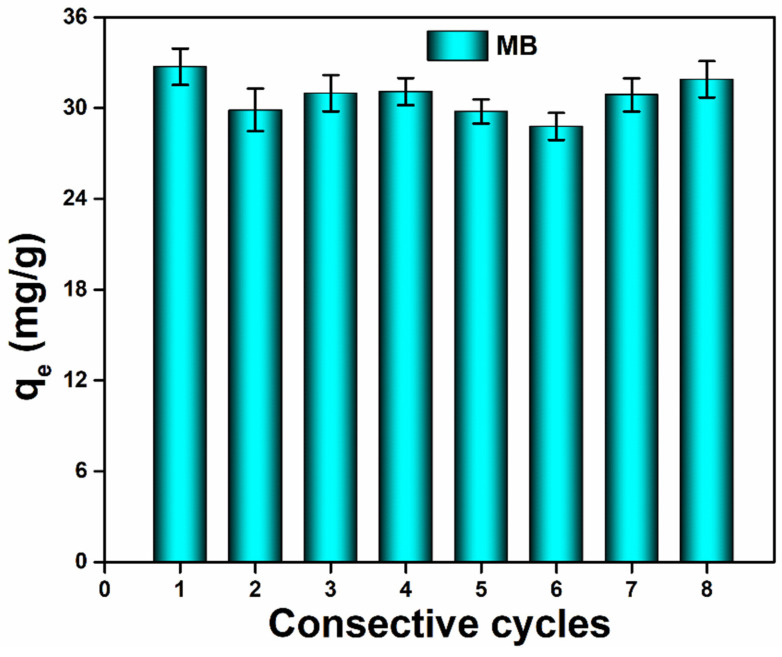
Relative photocatalytic capacities and restoration behavior of the fabricated PCGC@Ag nanofibrous membrane against methylene blue (MB) towards ambient for consecutive cycles.

In addition, significant degrees of stability and extents of recovery can be anticipated with the widespread use of nanofibrous membranes for the treatment of wastewater. We examined the effective water treatment behavior of recently developed nanofibrous materials. We looked into of the removal of MB and Rh-B using PCGC and PCGC@Ag nanofibrous materials. The results indicate that the degree of catalytic activity toward MB remained at about 22.78 mg/g (99.9% in the first catalytic process) after eight consecutive cycles, as shown in Figure 6. Alternatively, it can be said that the PCGC@Ag nanofibrous membrane maintains its structural properties after eight cycles of successive elution and provides excellent consistency and recycling of wastewater [41,42]. Because the electrospun membrane assists the Ag catalysts, the PCGC@Ag membrane exhibits exceptional photocatalytic activity. In conclusion, the fabricated nanofibrous membrane may be used for various industrial applications.

## 4. Conclusions

In this study, we report the fabrication of nanofibrous membranes (PLA/CMC/GO-*f*-COOH) via electrospinning and thermal treatment in order to achieve successful cross-linking. The composite nanofibrous membranes (PLA/CMC/GO-*f*-COOH@Ag) were prepared by dipping them into AgNO_3_ solution in order to anchor silver nanoparticles to enhance their stability for catalytic dye degradation applications. These nanofibrous membranes had high surface area and strong charge due to GO π–π stacking and the negative carboxyl group. The enhanced negative charge enables the targeting of diffused dyes, promoting their adsorption via a strong electrostatic interaction. The pore size also has an impact on water treatment. The different nanofibrous membranes have different pore sizes, whereby PCGC = 21.63 µm, PCGC@Ag = 32.51 µm and PLA = 39.75 µm, and achieving the optimum pore size, such as by a composite nanofibrous membrane, i.e., PCGC@Ag, is essential for performing water treatment. Therefore, the average pore size affects pseudo first- and second-order degradation rate, with the R^2^ values of PCGC@Ag (R^2^ = 0.989832, R^2^ = 0.998738) being higher than those of PCGC (R^2^ = 0.973594, R^2^ = 0.994392), which confirms that the dye degradation performances of PCGC@Ag are better than those of PCGC for methylene blue and Rh-B, respectively. The catalytic degradation of methylene blue by PCGC@Ag nanofibrous membranes offers substantial catalytic activity with excellent efficiency after eight cycles of catalytic degradation. Hence, the fabricated nanofibrous membrane has potential as a multifunctional and eco-friendly system with photocatalytic dye degradation properties for water treatment applications.

## Figures and Tables

**Table 1:** Summary of the photocatalytic and degradation kinetics studies.

Dye Name	Sample Name	Pseudo First Order			Pseudo Second Order		
		qe (mg/g)	R^2^	K_1_ (min^−1^)	qe (mg/g)	R^2^	K_1_ (g/mg min)
MB	PCGC	3.19	0.983581	0.0442	2.85	0.978329	0.0125
	PCGC@Ag	3.37	0.992917	0.1445	2.49	0.989839	0.1072
Rh-B	PCGC	23.93	0.973594	0.01149	33.62	0.994392	2.07 × 10^−4^
	PCGC@Ag	22.78	0.989832	0.07376	21.49	0.998738	1.20 × 10^−2^

## Data Availability

The data contained within the article.
